# Genetic Polymorphisms and Posttraumatic Complications

**DOI:** 10.1155/2010/814086

**Published:** 2011-01-11

**Authors:** Wei Gu, Jianxin Jiang

**Affiliations:** ^1^State Key Laboratory of Trauma, Burns and Combined Injury, Institute of Surgery Research, Chongqing 400042, China; ^2^Research Institute of Surgery, Daping Hospital, Third Military Medical University, Daping, Chongqing 400042, China

## Abstract

Major trauma is the leading cause of death in young adults. Despite advances in prehospital system and treatment in hospital, mortality rates have not improved significantly over the past decades. Victims of severe injuries who survive the initial hours have great risk for additional life-threatening complicaitons, including uncontrollable infection (sepsis) and multiple organ dysfunction syndrome (MODS). Single nucleotide polymorphisms (SNPs) have been shown to affect susceptibility to the course of numerous diseases. Accumulating evidence suggests that genetic backgrounds also play important roles in posttraumatic complications. Genetic polymorphisms may become powerful biomarkers for diagnosis and prognosis of trauma-induced complications. Recent advances in studies on associations between genetic polymorphisms and sepsis or MODS have led to better understanding of posttraumatic complications. Here we summarise recent findings on genetic variations in molecules of the innate immune system and other systems as well as their connection with susceptibility to posttraumatic complications.

## 1. Introduction

Major trauma is the leading cause of death in young adults and the third most common cause of death overall [1]. Advanced prehospital systems now deliver victims to hospitals where imminent threats to life, including airway compromise, hypoxia, hemorrhage, and intracranial hypertension are identified and controlled within few hours. However, victims of severe injuries who survive the initial hours have great risk for additional life-threatening complications, which include uncontrollable infection (sepsis) and multiple organ dysfunction syndrome (MODS), which remains a worldwide problem that is the leading causes of intensive care unit mortality [2, 3]. The causes of sepsis/MODS are numerous (e.g., age, sex, and injury), but the reasons why certain individuals develop sepsis/MODS while others following similar trauma insults do not are not well understood. There is ample evidence in the literature that gene-host and gene-environment interactions may play a large role in the morbidity and mortality associated with these complications. 

Early evidence favoring a role for genetic differences in trauma outcomes from a study conducted in animal subjects. Radojicic et al. [4] reported significant differences of their resistance to mechanical, thermal, and radiation trauma among four inbred strains of mice (AKR, BALB/c, CBA, and C57BL/6). Studies from our laboratory also showed that the mortality of C57BL/6 mice was significantly lower than BALB/c mice after injured by blast wave [5]. In an early epidemiological study, a strong association between death from infection in adoptees and their biological, but not adoptive, parents, also suggested a genetic influence on the risk for and outcome from infection [6]. Subsequent to these initial observations, numerous studies have identified that genetic factors play an important role in the pathogenesis of complications after trauma. 

Genetic variations include insertions, deletions, duplications, or single nucleotide polymorphisms (SNPs). Researchers have found that genetic polymorphisms might affect clinical phenotype by altering the function of the encoded protein, either by changing the structure of this protein or by modifying the expression of a gene. Furthermore, in contrast to protein biomarkers that may be transiently expressed during disease pathogenesis, gene polymorphisms also do not vary in response to underlying illnesses, and may be predictive indicators of disease susceptibility. Therefore, a genetic approach to assessing individual reaction to severe trauma is attractive since genotype can be easily determined from peripheral blood with minimal risk. Since trauma is sporadic, genetic association studies are more useful tools in investigating its possible relationships with gene polymorphisms than linkage studies. Recent advances in genotyping technologies have greatly expanded the number of studies that can test possible associations between gene polymorphisms and certain phenotypes. This paper aims to critically review evidence on the role of genetic polymorphisms in the pathogenesis of posttraumatic sepsis and MODS, based on association studies conducted primarily in human. We discuss the advantages and limitations of present studies and explore the application of microarray and other technologies to this important clinical and scientific problem.

## 2. Methodology of Genetic Association Studies on Trauma

In general, there are two approaches to perform genetic association studies: the candidate-gene approach and the genomewide approach (also known as genomewide association studies, GWAS). The completion of the HapMap project and development of high-density genomewide SNP arrays have enabled GWAS for many human complex diseases, such as Type II diabetes [7], prostate cancer [8], pulmonary sarcoidosis [9], and asthma phenotypes [10]. Unbiased GWAS have provided important insight to novel susceptibility genes for disease. However, no GWAS studies have not yet been published for complications after trauma, may be because of the expensive cost and complex etiology and multifactorial nature of trauma. In the candidate-gene approach, a few SNPs are genotyped on a gene of interest, which is chosen based on a biological hypothesis for the disease. The knowledge of the pathophysiology of sepsis and MODS has directed the search for candidate genes relevant to these clinical syndrome [11]. Genes of the pattern-recognition receptors (PRRs) system, complement system, coagulation system, cytokines, and signal-transduction molecules contribute to the list of candidate genes for sepsis and MODS that show genomic variation.

## 3. Genetic Polymorphisms Assisted with Complications after Trauma

Using the methods described above, various candidate susceptibility genes have been identified. And a number of investigations have evaluated the role of functional polymorphisms in case-control investigations. Reviewed below are some of the genes that have been investigated for association with the outcome after trauma.

## 4. Pattern-Recognition Receptors System Polymorphisms 

Pattern recognition receptors have recently been discovered and a concept has arisen focusing on microbial “patterns” associated with pathogens. Numerous studies have been performed to link these receptors to disease phenotypes, including sepsis. TLRs are key cellular receptors for initiation of the inflammatory response that recognise invading microbes and are an integral component of the innate immune system [12, 13]. Because of their importance in both the innate immune response and the induction of adaptive immunity, TLRs are currently at the centre of both basic research and drug development. Two groups of TLRs exist: one group is expressed on the surface of immune cells and recognises components of microbial cell walls such as lipopolysaccharide (LPS) of Gram-negative bacteria (TLR4) and lipopeptides (TLR2/TLR1 or TLR2/TLR6) or microbial proteins such as flagellin (TLR5) and protozoan profilin (TLR11); the other group of TLRs is expressed within the cell and recognises certain nucleic acids, such as single-stranded or double-stranded RNA (TLR7/TLR8 and TLR3, resp.), or CpG-rich DNA (TLR9) in specific cellular compartments. The relationship between gene polymorphisms in TLR1, TLR2, TLR4 and sepsis, and MODS has been well studied (Table 1). Although Arg753Thr in TLR2 gene has been shown to be associated with Gram-positive infections [14, 15], it was not existing in Chinese population. Our studies identified that another tagging SNP in TLR2 gene (19216T/C) was associated with cytokine production and seemed to confer an increased risk of sepsis and MODS after trauma [16]. We also found that −2242T/C and 11367G/C were two functional SNPs in TLR4 gene and may be related to sepsis morbidity [17–19]. There are comolecules associated with TLR4 sensing, including MD-2, CD14 and LPS-binding protein (LBP). A polymorphism in MD-2 promoter (−1625C/G) influenced MD-2 promoter activity and expression in vitro, and showed clinical influence in sepsis after trauma [20]. Although studies regarding CD14 have gotten conflicting results, our study identified that −159C/T and −1145G/A are both related to posttraumatic complications and had synergistic effects [21]. LBP SNPs were studied as part of association studies and there were also conflicting results with regard to sepsis [22, 23]. However, one recent cohort and functional study in our laboratory could confirm an increased risk of infection with Pro436Leu in LBP gene.

## 5. Gene Polymorphisms in Signal Transduction System

Intracellular signal transduction involves several steps, including numerous adaptor molecules and intracellular kinases. However, Gene polymorphisms in these genes were seldom studied. The Ser180Leu SNP in TIRAP/Mal gene was shown to be associated with invasive pneumococcal infection [36]. IRAK-1 1595T/C was also associated with increased risk for sepsis [35]. Two functional mutations in IRAK-4 were found in a patient with recurrent bacterial infections [34]. A study focused on NF-*κ*B activity in invasive pneumococcal identified two SNPs (rs3138053 and rs2233406) associated with susceptibility of infection [37], referring a key role for the transcription factor NF-*κ*B in the host response to infection.

## 6. Cytokine Polymorphisms

During sepsis, there is a full-blown, systemic activation of immune responses. As a result, sepsis is accompanied by a markedly imbalanced cytokine response (known as a “cytokine storm”), which converts responses that are normally beneficial for fighting infections into excessive, damaging inflammation. As effectors, cytokines released from immunocompetent cells play major role in the inflammatory response to infection. As a result, a number of variations in cytokine genes have been reported in gene association studies.

TNF*α* is a prototypical proinflammatory cytokine. The relationship between TNF*α*/−308 SNP and sepsis has been studied extensively. An association with sepsis severity and outcome following different inflammatory insults was found repeatedly, with a tendency towards increasing levels of TNF*α* and therefore a stronger inflammatory response [52–54]. However, these results have not been confirmed in other studies [55, 56]. Another important proinflammatory cytokine is interleukin (IL)-1 (isoforms *α* and *β*). An IL-1*α* polymorphisms was described for intron 6 (“variable number tandem repeats”, 46 bp) but this variation of IL-1*α* failed to show an association with sepsis [38]. However, our study found that polymorphisms in the IL-1*β* gene was associated with worse outcome in severe trauma patients [39, 40]. Besides these, polymorphisms in other cytokine genes, including IL-6, IL-10, TNF*α*, TNF*β*, IFN-*γ*, and MIF were studied (Table 1). Since these genes are form crosstalks in the pathogenesis of sepsis, we further studied the synergetic effects of 13 SNPs in 9 cytokines and found that polymorphisms of IL-1*β*/-1470, IL-1*β*/-511, IL-1*β*/-31, IL-4/-589, IL-6/-572, IL-8/-251, IL-10/-819, and TNF*α*/-308 are susceptibility loci for the development of sepsis and organ dysfunction in major trauma patients. Patients with more than four risk alleles of the eight SNPs had more than 50% sepsis morbidity and more severe organ dysfunction [68].

## 7. Coagulation System Polymorphisms

In the clinical setting of sepsis, dysregulation of the coagulation cascade results in major complications [69]. The extent of activation of the coagulation cascade during sepsis can range from an insignificant level to the occurrence of disseminated intravascular coagulation (DIC). Therefore, inherited variations associated with infections and sepsis have been described for coagulation factors such as plasminogen activator inhibitor-1 (PAI-1), thrombin-activatable fibrinolysis inhibitor (TAFI), fibrinogen, and factor 5 (Table 1). Studies on genetic variation in PAI-1 have been associated with morbidity of septic shock in meningitis patients [63]. For Thr325Ile in the TAFI gene, the outcome for meningitis was negatively influenced in homozygous carriers [64]. Furthermore, a protective haplotype GAA has been described in fibrinogen gene [66]. For Factor V/R506Q, the heterozygous carriers have smaller risk of sepsis [65].

## 8. Future Directions

Trauma is the forth major reason of morbidity and mortality all over the world, with associated great societal costs every year. The prevention and treatment of injury-induced sepsis and MODS are an international priority. It is not difficult to predict that the number of genetic studies concerning complications after major trauma will continue to increase. At the same time, platforms for genetic sequencing and expression analysis, as well as international databases such as HapMap, have been greatly improved in recent years. It will certainly facilitate genetic synchrony of genotypic and phenotypic data using enormous numbers of trauma patients. 

Although ongoing hypothesis-testing approaches will continue to bring important insights, they might be limited in their ability to provide a coherent, integrated view of genetic background in trauma patients. GWAS has been suggested as a potential powerful tool for future genetic predisposition studies. However, we emphasise that this systematic, whole-genome approach, that always ignores important information due to low power, must complement, not replace, the traditional single-gene approach. 

Taking into account the complexity of the pathogenesis of posttraumatic complications, we and others seek to interfere the synergetic effects of polymorphisms in mutiple genes and better comprehend the pathophysiology of multiple interactions in sepsis and MODS after trauma. With genotyping techniques becoming more and more advanced and with the cost decreasing, larger sample studies from multiple centers in the future may lead to a clearer picture of the individual variation in response patterns leading to a change in susceptibility of complications after trauma.

## Figures and Tables

**Table 1:** Effects of gene polymorphisms on sepsis or MODS.

Gene	Chrome location	Variation	Study size	Functional effects	Clinical effects on sepsis or MODS	Reference
Pattern-recognition receptors						

TLR1	4p14	−7202A/G (rs5743551)	999	Cytokine production and TLR1 expression	Sepsis, organ dysfunction and death (ODD), sepsis related acute lung injury (ALI)	[24]
		I602S		IL-6 production and NF-*κ*B signalling		[25]

TLR2	4q32	−16933T/A	252		increased prevalence of sepsis and with Gram-positive bacteria	[15]
		Arg753Thr	91	staphylococcal infections	Association with Gram-positive infection	[14, 15]
		19216T/C (rs3804099)	410	Cytokine production	Association with sepsis	[16]

TLR4	9q33. 1	896A/G	598		Association with decreased risk of complicated sepsis	[26]
		Asp299Gly, Thr399Ile	307/319/116		sepsis; Gram-negative septic shock; Conflicting results	[27–29]
		−2242T/C	303	Cytokine production and promoter activity	Association with sepsis and MODS	[17]
		11367G/C	132	gene expression	Association with sepsis and MODS	[18, 19]

LBP	20q11.23	Cys98Gly, Pro436Leu and 1683T/C	454/1215	higher median basal serum LBP levels	Gender-specific association with sepsis. Bacteraemia after stem cell transplantation and death from Gram-negative bacteraemia	[22, 23]

CD14	5q31.1	−159C/T(−260C/T), −1145G/A	293/85/319/ 116/252/430	higher monocyte mCD14, but not sCD14 expression	higher mortality; higher sepsis morbidity. Conflicting results	[15, 21, 28–32]

MD-2	8q21.11	103G/A (Thr35Ala)	20		Decreased cytokine release. No influence on sepsis studied	[33]
		−1625C/G	105	MD-2 promoter activity, MD-2 expression	Association with sepsis and MODS after trauma	[20]

Signal transduction						

IRAK-4	12q12	877C/T, 620-621/AC deletion	1	IRAK-1 kinase activity	Severe infections in childhood	[34]

IRAK-1	Xq28	1595T/C (haplotype)	155	nuclear levels of NF-*κ*B	Increased mortality in sepsis	[35]

TIRAP/Mal	11q24.2	Ser180Leu (rs8177374)	6106		Heterozygous carriers associated with infectious disease	[36]

I*κ*B	14q13	rs3138053, rs2233406	1060		Association with invasive pneumococcal	[37]
Cytokines						

IL-1*α*	2q14	46 bp VNTR			No association with sepsis	[38]

IL-1*β*	2q14	−31C/T, −511C/T	60/276/238	Higher production of IL-1*β*	Association with sepsis; Higher mortality in homozygous carriers with meningococcal sepsis. Conflicting results	[38–41]

IL-1RN	2q14.2	intron 2, VNTR	78		Higher mortality in homozygous carriers	[38, 42]

IL-6	7p21	−174G/C	69/288/293	Baseline of C-reactive protein	C-allele confers increased risk of shock	[30, 43–45]
		−572C/G	453	IL-6 production from leukocytes after LPS ex vivo	Sepsis in major trauma patients	[46]

IL-10	1q31-32	−592C/734G/3367G, −1082G/A	550/33	interleukin-10 production	Association with sepsis from pneumonia, increased mortality in severe sepsis	[47–51]

TNF*α*	6p21.3	−308G/A	1321/197		Association not clear. Early studies suggest higher risk when homozygous	[52–60]

TNF*β*	6p21.3				Association not clear	[56]

IFN-*γ*	12q14	CA repeat	61		Association with sepsis	[61]

MIF	22q11.23	−173G/C, −794 CATT repeat		MIF RNA and protein levels from mononuclear cells stimulated with bacteria	Influence on sepsis in African–Americans	[62]

Coagulation system						

PAI-1	7q21.3-q22	4G/5G	50	Increased gene transcription in cell lines in vitro and with increased PAI-1 concentrations in carriers in vivo	Higher rate of septic shock in meningitis	[63]

TAFI	13q14.11	Thr325Ile	50		Higher risk of death in meningitis	[64]

Factor V	1q23	R506Q	3894		Smaller risk of sepsis (heterozygous)	[65]

Fibrinogen	4q28	−854G/A, −455G/A, and +9006G/A. −148C/T	631/73	higher fibrinogen levels	Haplotype GAA was associated with a significantly lower 28-day mortality	[66, 67]
